# Nutrient Deficiencies Impact on the Cellular and Metabolic Responses of Saxitoxin Producing *Alexandrium minutum*: A Transcriptomic Perspective

**DOI:** 10.3390/md21090497

**Published:** 2023-09-18

**Authors:** Muhamad Afiq Akbar, Nurul Yuziana Mohd Yusof, Gires Usup, Asmat Ahmad, Syarul Nataqain Baharum, Hamidun Bunawan

**Affiliations:** 1 Department of Microbiology, Faculty of Biotechnology and Biomolecular Sciences, Universiti Putra Malaysia, Serdang 43400, Selangor, Malaysia; 2 Aquatic Animal Health and Therapeutics Laboratory, Institute of Bioscience, Universiti Putra Malaysia, Serdang 43400, Selangor, Malaysia; 3 Institute of System Biology, Universiti Kebangsaan Malaysia, Bangi 43600, Selangor, Malaysia; nataqain@ukm.edu.my; 4 Department of Earth Science and Environment, Faculty of Science and Technology, Universiti Kebangsaan Malaysia, Bangi 43600, Selangor, Malaysia; yuziana@ukm.edu.my (N.Y.M.Y.); gires@ukm.edu.my (G.U.); 5 Department of Biological Sciences and Biotechnology, Faculty of Science and Technology, Universiti Kebangsaan Malaysia, Bangi 43600, Selangor, Malaysia; asmat@ukm.edu.my

**Keywords:** saxitoxin, transcriptomics, gene set enrichment analysis, harmful algae blooms

## Abstract

Dinoflagellate *Alexandrium minutum* Halim is commonly associated with harmful algal blooms (HABs) in tropical marine waters due to its saxitoxin production. However, limited information is available regarding the cellular and metabolic changes of *A. minutum* in nutrient-deficient environments. To fill this gap, our study aimed to investigate the transcriptomic responses of *A. minutum* under nitrogen and phosphorus deficiency. The induction of nitrogen and phosphorus deficiency resulted in the identification of 1049 and 763 differently expressed genes (DEGs), respectively. Further analysis using gene set enrichment analysis (GSEA) revealed 702 and 1251 enriched gene ontology (GO) terms associated with nitrogen and phosphorus deficiency, respectively. Our results indicate that in laboratory cultures, nitrogen deficiency primarily affects meiosis, carbohydrate catabolism, ammonium assimilation, ion homeostasis, and protein kinase activity. On the other hand, phosphorus deficiency primarily affects the carbon metabolic response, cellular ion transfer, actin-dependent cell movement, signalling pathways, and protein recycling. Our study provides valuable insights into biological processes and genes regulating *A. minutum*’s response to nutrient deficiencies, furthering our understanding of the ecophysiological response of HABs to environmental change.

## 1. Introduction

*Alexandrium minutum* can contribute to harmful algae blooms (HABs) in marine ecosystems. In addition, this species is known to produce saxitoxin, a potent neurotoxin that can be deadly to humans. HABs are influenced by various factors, such as physical conditions (currents), chemical conditions (nutrient presence), and biological factors (dinoflagellate species). Among these, nutrients are considered key drivers in HAB formation, particularly nitrogen, and phosphorus, which also play a crucial role in regulating dinoflagellate toxin production [1,2,3,4,5]. For instance, field measurements showed that high nitrogen and phosphorus concentrations have been associated with blooms of the harmful dinoflagellate taxa [4,5]. 

Nitrogen is an essential component in saxitoxin; therefore, the biosynthesis of saxitoxin in dinoflagellates is influenced by nitrogen level [6,7]. *A. minutum* has been observed to experience decreased growth and saxitoxin biosynthesis under nitrogen stress [8]. Phosphorus deficiency, conversely, has been associated with increased saxitoxin biosynthesis in most dinoflagellates, including *A. minutum* [8,9]. Thus, studying the metabolic reactions that occur under nitrogen and phosphorus stress is critical for understanding the mechanisms of HAB formation and toxin production. 

Nitrogen constitutes up to 14% of the dry weight of dinoflagellate cells. The nitrogen taken up can be assimilated into active biochemicals, which can be used intracellularly for various metabolic and physiological processes [7]. Nitrogen availability also affects cell metabolism, growth, biochemical composition, photosynthesis, and other cellular activities involving cellular physiological activity. Nitrogen deficiency inhibits cell growth, alters pigment composition, lowers photosynthetic energy uptake and photosynthetic efficiency, and can also trigger cyst formation by certain dinoflagellate species [10,11,12].

Phosphorus, similarly, is essential for dinoflagellate growth and is required for cellular structure, metabolism, energy storage, cell signaling, and biochemical regulation [13,14]. However, in marine ecosystems, dissolved inorganic phosphorus (DIP) is often in limited supply [13]. In response to low phosphorus availability, phytoplankton species have developed various strategies such as altering phosphorus transport protein activity, using dissolved organic phosphorus (DOP) as a source of phosphorus, altering cell membrane composition, and bypassing phosphorus uptake processes in pathways such as glycolysis and phospholipid recycling [11,15]. 

Our previous study described a comprehensive gene catalog for *A. minutum* that highlights various metabolic pathways and critical adaptations to the marine environment [16]. *A. minutum* might exhibit physiological adaptability in response to a variety of environmental challenges, including nutrition deprivation [8,9,17,18]. Previously, Meng et al. [18] *A. minutum* is capable of employing ecological adaptation strategies when faced with stress conditions. These strategies encompass shifts in trophic modes and related physiological responses. Despite recent advancements, our understanding of how significant nutrient deficiencies impact the cellular and metabolic functions of *A. minutum* remains limited, particularly considering that dinoflagellate metabolism is regulated through a complex interplay of transcriptional and post-translational mechanisms [17,18]. This study uses transcriptome analysis to determine the effects of nitrogen and phosphorus stress on *A. minutum* to identify biological effects resulting from changes in the gene expression of *A. minutum*. This study demonstrated gene expression profiles under nutrient-replete, nitrogen-deficient, and phosphorus-deficient conditions. In this study, we employed gene set enrichment analysis, a more sensitive approach for interpreting and analyzing transcriptional changes, in contrast to the over-representation analysis used in previous studies [18,19,20]. These will provide important insights into the mechanisms *A. minutum*, and other dinoflagellates might use to overcome nitrogen and phosphorus deficiencies in the marine environment.

## 2. Results and Discussions

### 2.1. Comparison of Unigene Expression Profiles A. minutum under the Induction of Nitrogen and Phosphorus Deficiency

**Figure 1:**
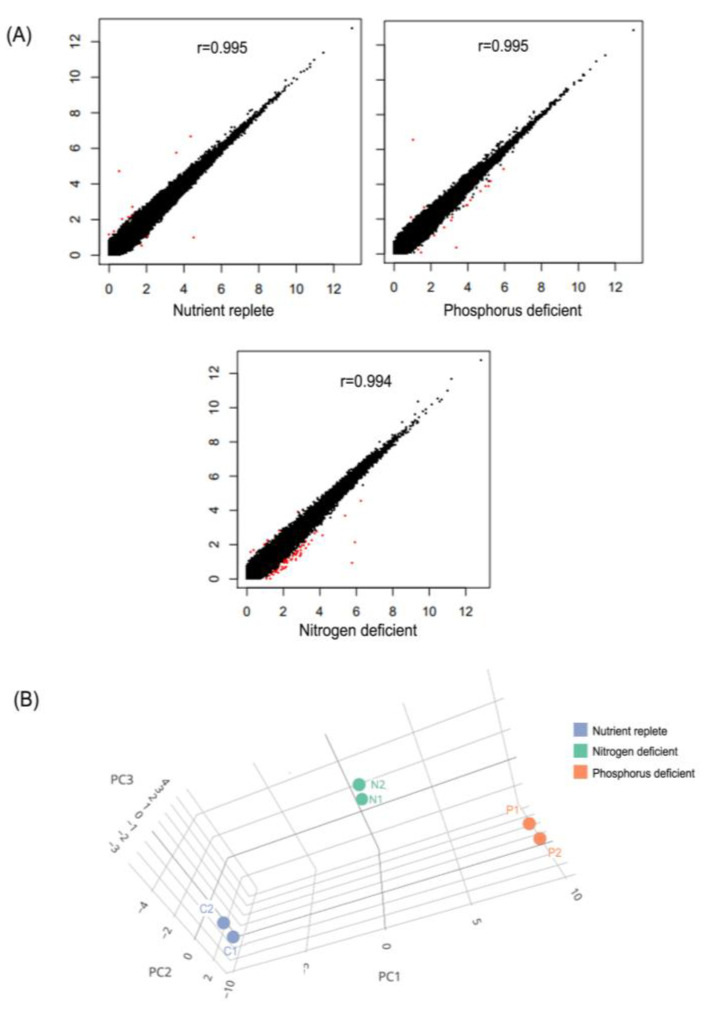
Comparison of unigene expression values between all samples. (**A**) Scatter plot comparing unigene expression values for biological replicates and (**B**) 3D PCA plot of unigene expression values for all samples.

To compare the *A. minutum* unigene expression levels in control samples and nutrient-deficient samples (nitrogen and phosphorus-deficient), readings of high-quality raw data from each sample were mapped to a total of 124,977 *A. minutum* unigenes and the expression of each unigene in different samples was estimated using the RSEM program. The expression reading value of each unigene is also expressed as a raw count value as well as a TPM value. Next, the reliability of the biological replicas used in this study was tested using the scatter plot comparison method (Figure 1A). A high correlation between the two biological repeat samples indicated a high level of repeatability, with the Pearson correlation coefficient R equal to 0.99 for all samples. This suggests that repeated biological samples produced acceptable data for further analysis. Finally, to evaluate the influence of nitrogen and phosphorus on the gene expression profile of *A. minutum*, this study adopted a multivariate analytical approach through principal component analysis (PCA). Figure 1B clearly shows the PCA plots of gene expression profiles from all six samples successfully differentiated into three groups in line with the experimental design.

**Figure 2:**
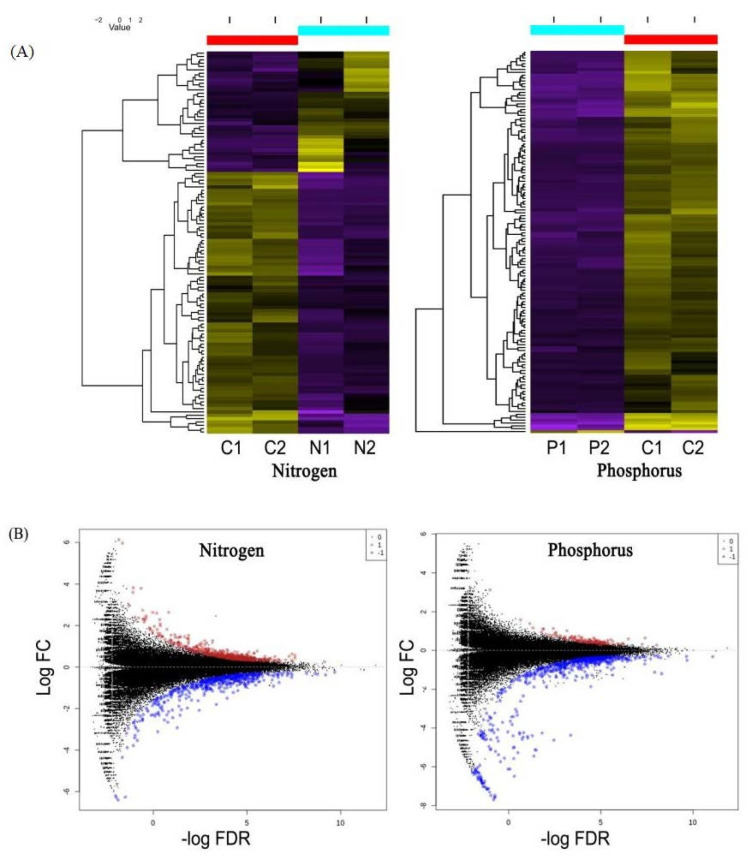
*A. minutum* unigenes expressed differently under the induction of nitrogen and phosphorus deficiency. (**A**) The heat map based on TPM log values (**B**) MA plot displays all DEGs. Red dots represent up-regulated DEGs, while blue dots represent down-regulated DEGs.

To identify unigenes that showed significant differences during nitrogen and phosphorus-deficient conditions, comparisons of normalized gene expression profiles using the TMM method were performed using the edgeR program. In total, 1812 unigenes had a threshold value of FDR < 0.05 for differently expressed genes (DEGs). Of these, 0.4% of the studied unigenes (538/124 977) were identified as a gene with a higher expression during nitrogen deficiency, and 0.4% of the studied unigenes (511/124 977) had a lower expression when compared with the control group. In the case of phosphorus deficiency, 0.1% of the studied unigenes (127/124 977) had a higher expression, while 0.5% of the studied unigenes (636/124 977) had a lower expression. The set of DEGs identified in this study was subsequently plotted as MA plots and heat maps (Figure 2). However, most of these unigenes do not have a protein sequence match on the NCBI database nr. The full list of DEGs identified in this study is available in Table S1.

### 2.2. GSEA Analysis of A. minutum under the Induction of Nitrogen and Phosphorus Deficiency

**Figure 3:**
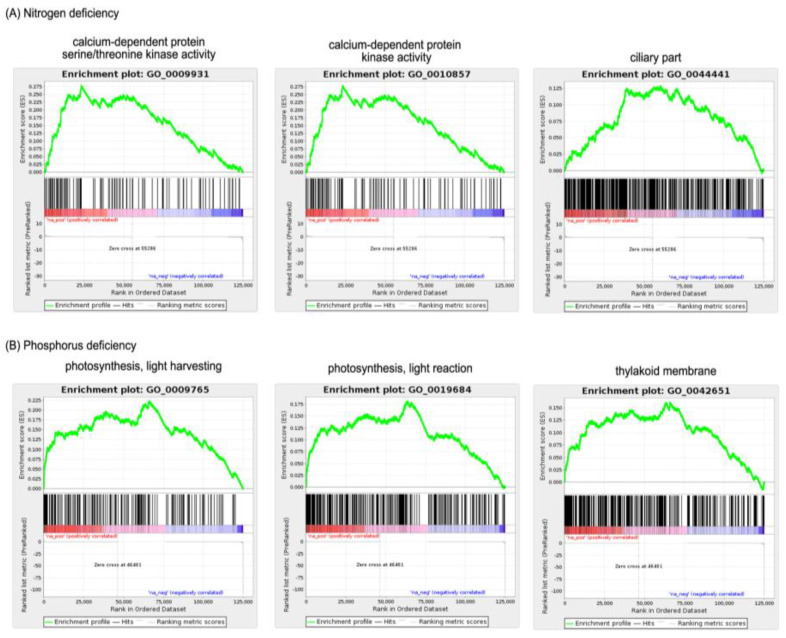
Top three enriched GO terms by *A. minutum* under the following conditions. (**A**) Nitrogen deficiency (**B**) Phosphorus deficiency.

Furthermore, an enrichment analysis of the GO term was performed to provide an overview of the biological functions and processes associated with DEGs. This study utilized the GSEA approach for enrichment analysis, which is known to be more effective and sensitive than other methods, such as the Fisher exact test and over-representative analysis (ORA). The GSEA approach analyzes the position and expression level of all unigenes in the study sample without being restricted by arbitrary threshold values such as FDR or DEG multiplier log changes, making it a more comprehensive and unbiased method for identifying enriched gene sets [19,20]. The results of GO enrichment analysis using the GSEA program successfully identified 702 GO terms which were all found to be enriched (all up-regulated) under the condition of nitrogen deficiency, while 1251 GO terms, of which 1246 were up-regulated, and 5 were down-regulated, were enriched when phosphorus was deficient with a threshold value of FDR < 0.25. The top three GO terms in terms of normalized enrichment score (NES) for each category are shown in Figure 3. A higher NES score indicates a gene shift in a particular GO term to one end on the gene ranking file, determining whether the GO term is up-regulated or down-regulated (via positive or negative NES values). A complete list of enriched GO terms identified in this study is in Table S2.

**Figure 4:**
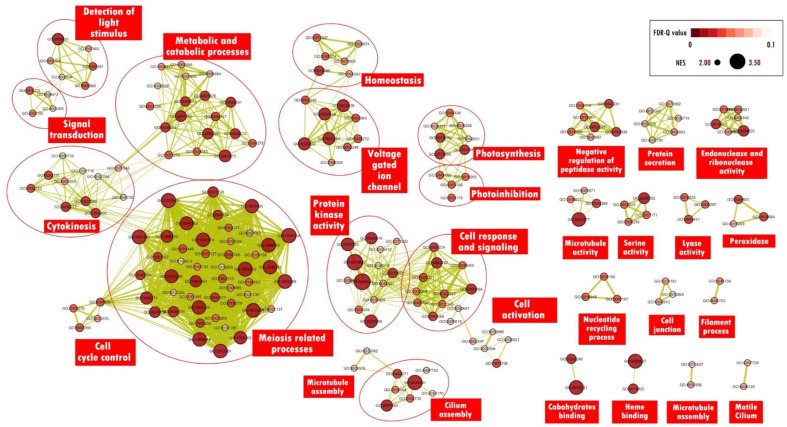
Enrichment map visualization for enriched GO terms by *A. minutum* under the induction of nitrogen deficiency. Each node corresponds to enriched GO terms in response to nitrogen deficiency, and the nodes’ size is related to the NES value for the enriched GO term. Edges (green lines) connect GO terms to shared genes, and line thickness is associated with the number of genes shared between the GO terms.

**Figure 5:**
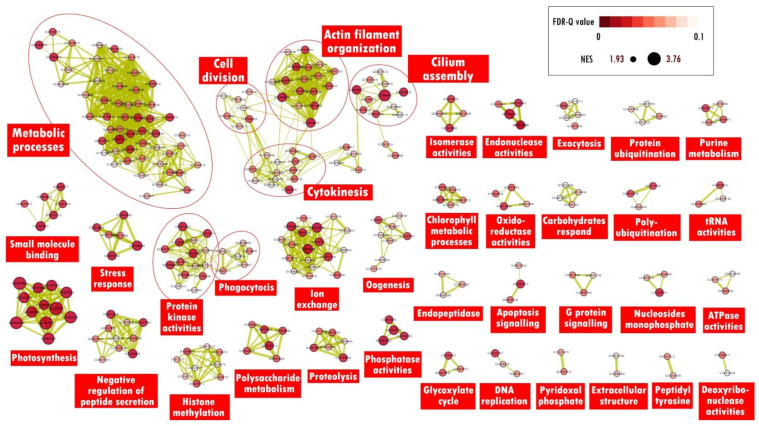
The Enrichment map visualizes the enriched gene ontology (GO) terms in response to phosphorus deficiency in *A. minutum*. Each node in the map represents an enriched GO term, with node size reflecting the normalized enrichment score (NES) for the term. The green edges indicate shared genes between the GO terms, with the thickness of the line indicating the number of shared genes.

GO enrichment analysis will often show the enrichment of several GO terms that are similar hence complicating the interpretation of data or study results. Concentrating or grouping GO terms according to general functions is one of the strategies used to facilitate the data interpretation process. The “enrichment map” function in Cytoscape software is a method to describe these enriched GO terms more simply. The results of the subsequent enrichment analysis can be viewed simultaneously in a single enrichment map. Only GO terms with an FDR threshold value < 0.1 were used to reduce repetition on the enrichment map. The resulting enrichment map for nitrogen-deficient samples displays 209 nodes and 1423 edges connected with an edge cut-off threshold value < 0.375. The “auto-annotate” function then classifies the mapped GO terms into 30 different clusters according to a shared general function. The largest cluster is the meiosis-related process cluster containing 47 enriched GO terms, followed by the metabolic and catabolic process cluster containing 18 enriched GO terms, and the protein kinase activity cluster containing 13 enriched GO terms. Next, for the enrichment of phosphorus-deficient samples, the resulting enrichment map displays 286 nodes and 1196 edges connected. The “auto-annotate” function then classifies the mapped GO terms into 42 different clusters according to a shared general function. The largest cluster is the metabolic process cluster containing 54 enriched GO terms, the ion transfer cluster containing 18 enriched GO terms, and the actin filament organization cluster containing 17 enriched GO terms. The enrichment map was generated to illustrate GO-enriched terms in *A. minutum* under nitrogen and phosphorus-deficient conditions (Figure 4 and Figure 5).

### 2.3. Enriched GO Term by A. minutum under the Induction of Nitrogen Deficiency

#### 2.3.1. Meiosis

A total of 47 GO terms related to the meiosis process were enriched in *A. minutum* under nitrogen deficiency (Figure 4). Some of these terms are related to the conversion of the cell cycle from mitosis to meiosis, a strategy used by dinoflagellates to form temporary cysts that can survive in unfavorable environments [7]. Homologs of meiosis protein mei2, DNA topoisomerase II, and serine/threonine kinase protein were found to contribute to the enrichment of GO:0045930. Mei2 is required for cells to migrate from mitosis to meiosis, while DNA topoisomerase II plays a key role in homologous chromosome separation during meiosis I and serine/threonine kinase proteins are involved in the termination of the mitotic cell cycle [21,22].

In addition, a previously unidentified unigene was found to contribute to the enrichment of GO:0045930. The unigene encodes a hypothetical protein of 151 amino acids and contains a forkhead-associated domain (FHA) sequence, which has been shown in past studies to be involved in DNA damage responses and cell cycle termination [23,24]. Mutations in the FHA domain of the fungus *Neurospora crassa* cause meiosis phenotypes and abnormal ascospore development [24]. These findings suggest that *A. minutum* may encode a novel protein involved in the process of cell division, consistent with the phenotypic characteristics of dinoflagellates, which have large and compact chromosomes [25].

#### 2.3.2. Catabolic Carbohydrate Metabolism

This study also identified several GO terms related to the catabolic carbohydrate metabolism process enriched in *A. minutum* under the induction of nitrogen deficiency. These results indicate that under the induction of nitrogen deficiency, *A. minutum* is also likely to mobilize carbon sources for cellular use as opposed to storing these carbon sources in the form of carbohydrates. Although carbohydrate metabolism processes under the induction of nitrogen deficiency have not been extensively studied in dinoflagellates, similar study results have been reported in several marine diatom species such as *Thalassiosira pseudonana* and *Phaeodactylum tricornutum* [26,27]. In fact, further LEA analysis also showed that the enrichment of GO terms in metabolic and catabolic process clusters was contributed to by unigenes encoding catabolic proteins such as alpha-amylase, exoglucanase, alpha-1,4 glucan phosphorylase, glucanase, and others. The alpha-amylase protein serves to catalyze the hydrolysis of glycoside bonds α-1 → 4 polysaccharides such as starch and glycogen while reducing cellular carbohydrate accumulation [28]. Wase et al. [28] found that Alpha-amylase protein expression by the microalgae *Chlamydomonas reinhardtii* also increased under the induction of nitrogen deficiency. Furthermore, the enrichment of the term GO related to glycolysis, “glycolytic process through fructose-6-phosphate” (GO:0061615), also strengthens the hypothesis that carbohydrate metabolism in *A. minutum* is catabolic under the induction of nitrogen deficiency. An increase in the rate of glycolysis under the induction of nitrogen deficiency in *A. minutum* indicates that carbon sources, such as intracellular carbohydrate storage, are transferred to central carbon metabolism. Hockin et al. [26] suggested that in nitrogen-restricted conditions, the degradation of intracellular carbon sources through catabolic metabolism is important for the nitrogen re-assimilation processes using the GS/GOGAT pathway as well as the urea cycle.

#### 2.3.3. Nitrogen Metabolism

Genes encoding the proteins involved in the GS/GOGAT cycle pathway contribute to the enrichment of the GO term “glutamine family amino acid metabolic process” (GO:0009064). These unigenes include genes that encode GS proteins and glutamate synthase-NADPH (GOGAT-NADPH) as well as glutamate synthase-ferredoxin (GOGAT-Fe). The GS protein catalyzes the fixation of ATP-dependent ammonium to the d-carboxyl group of glutamate to form glutamine. Next, the GOGAT protein catalyzes one molecule of glutamine and one molecule of 2-oxoglutarate to form two molecules of glutamate [29]. Thus, under the induction of nitrogen deficiency, *A. minutum* is likely to increase the activity of GS/GOGAT pathways to increase ammonium assimilation as a result of some cellular catabolic activity to compensate for nitrogen deficiency. In fact, some marine diatom species, such as *T. pseudonana* and *P. tricornutum*, also show increased expression of genes/proteins involved in the GS/GOGAT pathway under the induction of nitrogen deficiency [26,30]. In addition, the *A. minutum* unigenes encoding the GDH protein also contributed to the enrichment of the same GO term. In addition to the GS/GOGAT pathway, GDH protein activity can also catalyze the production of glutamate from ammonium and 2-oxoglutarate without the need for ATP [29].

#### 2.3.4. Ion Homeostasis

Ion channels are important components in cell signaling and are involved in environmental adaptation, cell growth and differentiation, cell movement, exo, and endocytosis [31,32]. Dinoflagellates are also likely to utilize ion channels in various cellular processes [33]. The enrichment map shows nine GO terms related to voltage-gated ion channels enriched in *A. minutum* (Figure 4). These results suggest that the movement of ions across the cell membrane is enhanced under nitrogen deficiency. This theory is supported by several enriched GO terms related to homeostatic processes. In dinoflagellates *Prorocentrum minimum*, the expression of genes involved in cellular calcium ion homeostasis increased under the induction of copper sulphate biocides, indicating that calcium ion homeostasis is a defense mechanism against stressful environments. However, the uncontrolled and excessive entry of calcium ions into the cell can also lead to oxidative stress and cell death [34]. Increased oxidative stress due to nitrogen deficiency has also been reported in dinoflagellates *P. lima* and the microalgae *Chlorella sorokiniana* [35,36]. In summary, the homeostasis of calcium ions by *A. minutum* under nitrogen deficiency is likely to serve as a signaling system to influence cellular processes to cope with this limitation. However, continuous nitrogen deficiency in the environment can lead to oxidative stress on cells. Further studies are needed to determine the detailed role of ion homeostasis processes in dinoflagellates under certain inductions.

#### 2.3.5. Protein Kinase Activities

In parallel with the increased entry of calcium ions into the cells of *A. minutum* discussed earlier, the activity of ion-dependent protein kinases was also enriched in *A. minutum* under nitrogen deficiency induction. LEA analysis revealed that *A. minutum* unigenes contributing to the enrichment of GO terms related to protein kinase activity are dominated by genes encoding calcium-dependent protein kinase, calmodulin-dependent protein kinase, and putative serine/threonine-protein kinase. Calcium ion binding catalyzes the activation of these proteins, which in turn react with other downstream proteins through phosphorylation activity [37]. Phosphorylation is a post-translational protein modification mechanism that activates or deactivates proteins and is a way to control signal transduction between metabolic pathways [38]. The fraction of proteins regulated using phosphorylation ranges from 10% to 60%, depending on the organism [39,40]. Protein phosphorylation activity also regulates diurnal rhythmic protein expression at the post-translational stage in dinoflagellates *L. polyedrum* [41,42]. Changes in protein phosphorylation activity under nitrogen deficiency induction have also been reported in plants and marine diatoms [39,43]. Thus, *A. minutum* is likely to regulate cellular processes in the post-translational stage to overcome nitrogen deficiency.

### 2.4. Enriched GO Term by A. minutum under the Induction of Phosphorus Deficiency

#### 2.4.1. Metabolic Processes

1. Glycolysis;

LEA analysis for the enrichment of the GO term “glycolytic process” (GO:0006096) includes the unigene encoding the pyrophosphate-fructose 6-phosphate 1-phosphotransferase (PFP) protein. This protein catalyzes an alternative glycolysis pathway which allows cells to bypass enzymatic reactions that require the use of phosphorus sources [44]. Unlike classical glycolysis pathways, these alternative glycolysis pathways are considered more advantageous in terms of cellular energy savings since these alternative pathways bypass ATP utilization [45]. In addition, the increased expression of PFP proteins is also associated in part with the mechanism of cell metabolic adaptation in a phosphorus-deficient environment during the occurrence of the HAB phenomenon [46]. Increased expression of genes/proteins involved in the glycolysis pathway under the induction of phosphorus deficiency has also been reported in most marine diatom species and some dinoflagellate species such as *Karlodinium veneficum* and *P. donhaiense* [47,48,49]. However, increased glycolysis rates in dinoflagellates under the induction of phosphorus deficiency is not a universal response, as there are also several past studies reporting decreased gene/protein expression associated with the glycolysis pathway [15,50]. Thus, in contrast to diatoms, alternative glycolysis pathways that are less dependent on organic phosphate sources are unlikely to be conserved by all dinoflagellate species [45].

2. Nucleic Acids Metabolism;

Nucleic acids comprise approximately 9% of dry-weight phosphorus [51]. Therefore, when there is a shortage of phosphorus sources for synthesizing new nucleic acids, cells will recycle phosphorus from existing nucleic acids in the most cost-effective way. *A. minutum* in this study showed enrichment of several GO terms related to nucleic acid metabolism. Unigene-encoding proteins such as phosphodiesterase, putative 5′-nucleotidase, phosphoglycerate kinase, and enolase with nucleic acid degradation activity contribute to the enrichment of GO terms related to nucleic acid metabolism. The recycling of phosphorus sources from nucleic acids has also been reported in several past studies [47,52]. Gene expression studies of the marine diatom *P. tricornutum* have also shown increased expression of the gene encoding the 5′-nucleotidase protein as early as 72 h after the induction of phosphorus deficiency [52]. Similar results have also been reported for the dinoflagellate *P. donghaiense* [49].

3. Amino acids metabolism.

The study by Müller et al. [53] indicated that *Lupinus albus* accumulated most free amino acids under phosphorus deficiency, with tryptophan showing the highest accumulation rate. Similarly, *A. thaliana* showed a significant increase in the accumulation of aromatic amino acid groups under phosphorus deficiency [54]. *A. minutum* is also likely to experience increased accumulation of free amino acids as some GO terms related to amino acid metabolism and biosynthesis are enriched under phosphorus deficiency. Enrichment of GO terms related to the biosynthesis of aromatic amino acid groups is contributed to by proteins such as fatty acid amide hydrolase, while the enrichment of the term GO related to the glutamine amino acid group is contributed to by unigenes encoding proteins involved in GS-GOGAT pathways and the urea cycle. The increase in expression of unigenes involved in the GS-GOGAT pathway and the urea cycle by *A. minutum* under phosphorus deficiency is likely due to increased intracellular ammonia, a by-product of protein degradation, which is a universal response of most organisms under phosphorus deficiency [44,47,55].

#### 2.4.2. Ion Transfer

The second largest cluster in the *A. minutum* enrichment map under phosphorus deficiency is the ion transfer cluster, with 18 GO terms mainly related to potassium and calcium transfer. Some plant species, including soy, have reported decreased intracellular potassium under phosphorus deficiency [56,57]. Potassium ions are known to play an important role in protein activation [58], but their function in eukaryotic photosynthetic organisms such as diatoms and dinoflagellates is still unclear. Calcium, on the other hand, is an essential macronutrient involved in cellular growth and development as well as functions as a signaling molecule, triggering cellular biochemical responses to stress [59,60]. Although the role of calcium ions in stress responses such as cold and hot temperatures and salinity has been studied, their role in signaling low nutrient stress, such as phosphorus deficiency, is not well explored, especially in dinoflagellates [60,61].

#### 2.4.3. Organization of Actin Filaments

The *A. minutum* enrichment map revealed a cluster of 17 GO terms related to actin filament organization enriched under phosphorus deficiency induction (Figure 5). Actin filaments participate in various protein–protein interactions and have been identified to regulate transcription processes, cell division, cell motility, and shape [62]. In dinoflagellates, actin filaments are also involved in bioillumination, chloroplast translocation, cell shape maintenance, and ecdysis [63,64,65,66]. LEA analysis of the enriched terms showed that proteins involved in cell motility processes, such as Fibronectin protein, casein kinase i isoform alpha, Catenin beta-1, and Myosin II, were encoded by contributing unigenes. This suggests that *A. minutum* cell motility is enhanced under phosphorus deficiency induction, allowing it to migrate to areas with optimal nutrient concentrations. Dinoflagellates such as *Akashiwo sanguinea* and *Prorocentrum micans* have been reported to undergo vertical migration to deep waters rich in phosphorus sources, which gives an adaptive advantage to some dinoflagellate species [67,68]. Thus, the increased motility of *A. minutum* under phosphorus deficiency induction might provide an advantage for this species over other phytoplankton groups to adapt to the marine environmental change.

#### 2.4.4. Cell Signaling Activity

GO terms related to mitogen-activated protein kinase (MAPK) signaling were enriched in response to phosphorus deficiency and concomitant with an increased influx of calcium ions acting as cell signaling molecules. MAPK is activated through the induction of cofactors, such as the presence of ions, and engages in responses to biotic and abiotic stresses [69]. MAPK signaling is also involved in the regulation of cell division processes and other reproductive activities through protein phosphorylation activities. In yeast, the organisms in which MAPK signaling is most studied, MAPK transduction signaling functions in modulating pheromone response, cell cycle control, and cell integrity [70,71]. In dinoflagellates, MAPK signaling pathways are reported to be involved in response to the induction of chemicals such as polychlorinated biphenyl (PCBs) and copper sulphate biocides [72,73].

In addition to MAPK signaling, G protein-coupled receptor (GPCR) pathways are crucial for signaling and responding to external stimuli such as hormones, nutrients, and phospholipids [74,75]. However, these pathways have not been well studied in eukaryotic photosynthetic organisms until recently. Mojib and Kubanek [76] demonstrated the presence of GPCR signaling pathways in several dinoflagellate species using a transcriptomic approach, and our study found that several GO terms related to the GPCR pathway were enriched under phosphorus deficiency. LEA analysis revealed that unigenes encoding major components of the GPCR pathway, such as G protein-coupled receptors, Ras-like, and cAMP-dependent protein kinase regulatory subunits, contributed to the enrichment of these GO terms. Previous studies have also shown that GPCR signaling plays a vital role in crosstalk activation of the MAPK signaling pathway [77,78].

#### 2.4.5. Methylation of Histones and Proteins

In response to environmental changes, epigenetic mechanisms reprogram gene expression to prepare cells for survival [79]. *A. minutum* enriched GO terms related to histone and protein methylation, a branch of epigenetic modification, under the induction of phosphorus deficiency. The role of histones in dinoflagellates is thought to be related to gene expression regulation, not chromosome arrangement. Several previous studies have shown unchanged gene expression encoding histone proteins throughout the cell cycle in some dinoflagellate species process [25,80]. The enrichment of histone methylation-related GO terms in this study supports the hypothesis that histone proteins regulate gene expression through epigenetic modifications in dinoflagellates. Histone methylation is also a component of the response to phosphorus deficiency in most plants [81,82].

#### 2.4.6. Ubiquitin Pathway

Our study showed that several GO terms related to the ubiquitin pathway were enriched under the induction of phosphorus deficiency and that all three major components of the pathway, including mitochondrial protein ubiquitin ligase activator, ubiquitin-conjugating enzyme E2-17 kDa, and putative E3 ubiquitin-protein ligase rbrA, contributed to this enrichment [83]. The ubiquitin pathway, which involves the covalent bonding of small ubiquitin proteins to target proteins and subsequent elimination of these pre-labeled target proteins via proteolysis by 26S proteasome proteins, is one form of post-translational regulation [84]. Proteolysis induced by the ubiquitin pathway plays an important role in basic cellular processes such as cell cycle and division regulation, stress response, and protein recycling processes [84,85,86]. Additionally, protein degradation through proteolysis produces free amino acids and ammonia as downstream products, which are important under phosphorus deficiency [87]. Further studies are needed to fully understand the role of ubiquitin pathways in the regulation of dinoflagellate cellular processes at the post-translational stage.

### 2.5. Implications of Nitrogen and Phosphorus Deficiency on Saxitoxin Biosynthesis

Our study found that the expression of the *A. minutum* unigene, which encodes homologs of proteins involved in saxitoxin biosynthesis, does not significantly change under conditions of phosphorus or nitrogen deficiency. These results are consistent with previous studies [3,6,88] stating that saxitoxin biosynthesis involves complex regulation at the post-transcriptional and post-translational levels. For example, Perini et al. [88] also found that the expression of homologs of two major genes in saxitoxin biosynthesis, sxtA, and sxtG, did not correlate directly with intracellular toxin changes in *A. minutum*.

The increased glycolytic activity during phosphorus deficiency in *A. minutum* may also result in the production of more acetyl-CoA and malonyl-CoA molecules, which are required to initiate saxitoxin biosynthesis. Under nitrogen deficiency, the low rate of saxitoxin biosynthesis is indirectly influenced by a decrease in cellular growth rate [89,90]. Additionally, during nitrogen deficiency, acetyl-CoA is redirected towards fatty acid synthesis, thereby reducing the pool of precursors available for saxitoxin biosynthesis in *A. minutum*.

## 3. Materials and Methods

### 3.1. RNA-Seq Data Retrieval

**Figure 6:**
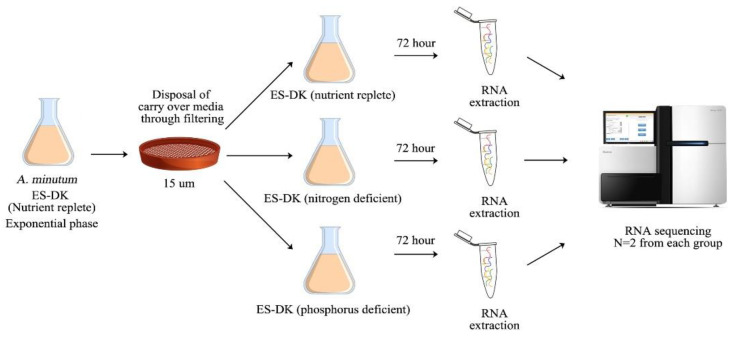
The experimental design adopted to induce nitrogen and phosphorus deficiency in *A. minutum.*

RNA-seq datasets used throughout this study were obtained from our previous research. These data can be downloaded from the NCBI Sequence Read Archive (SRA) Database under the BioProject id: PRJNA914202 [16]. In brief, *A. minutum* cells were grown in nutrient-replete ES-DK media until they reached the exponential phase before being transferred using a 15 m mesh filter to new ES-DK media lacking nitrogen/phosphorus components to induce nitrogen/phosphorus limitation in *A. minutum* cultures (Figure 6). Then, the RNA was harvested from *A. minutum* cells that were starved of nitrogen and phosphorus for 72 h. A HACH DR2800 spectrophotometer was used to confirm the growth media’s lack of nitrogen/phosphorus. The complete annotation pipeline for *A. minutum* transcriptome was described in our previous study [16]. A complete annotation of *A. minutum* unigenes is available in Table S3.

### 3.2. Estimation of Unigene Expression Levels

**Figure 7:**
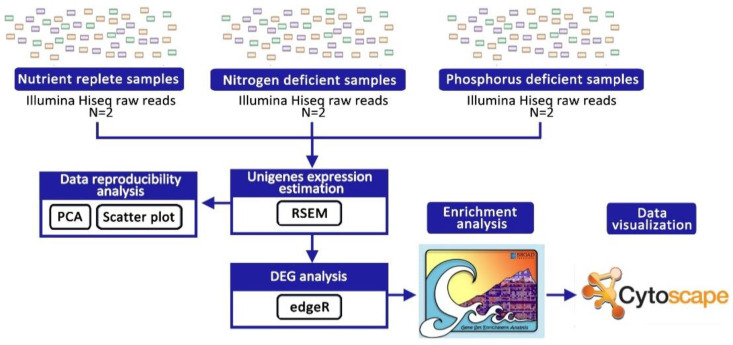
Workflow for transcriptomics data analysis used in this study.

The expression of each *A.minutum* unigene that was assembled in our previous study was calculated using the RNA-Seq by Expectation-Maximization (RSEM) program [91] (Figure 7). The RSEM program will first re-map high-quality raw data from each sample (two control samples, two nitrogen deficiency samples, and two phosphorus deficiency samples) to assemble unigene sequences using algorithms from the bowtie2 program [92]. Next, the RSEM program will use the Expectation-Maximization algorithm to identify each unigenes’ expression level. The expression reading value of each unigene is also displayed in the raw count value and the TPM value (Transcripts Per Million). TPM normalization aims to reduce the bias in unigene expression values present due to differences between unigene lengths [91].

Next, the raw count values for each unigenes were then used to generate scatter plots using the script “PtR compare_replicates” from the Trinity package to test the reliability of the biological replicates in this study (Haas et al. 2013). Finally, a Principal Component Analysis (PCA) plot was generated using the TCC-GUI program [93].

### 3.3. Differently Expressed Genes Analysis

An analysis to identify differently expressed genes (DEGs) was performed using the EdgeR program [94]. Using this program, the raw count values for each sample were normalized using the Trimmed mean of M-values (TMM) method in advance to minimize the bias resulting from the differences in the size of raw data for different samples. In this study, the EdgeR program was used with quasi-likelihood test parameter settings and glmQLFit to reduce the potential for false positive rates and increase the statistical power in detecting outlier values. The Benjamini–Hochberg method was used to calculate the false discovery rate (FDR) of unigenes expressed differently. FDR values < 0.05 were considered significant outcomes. The unigene expression levels expressed differently as per the TPM unit were then plotted as a heat map using the script “analyze_diff_expr.pl” in the Trinity program package [95].

### 3.4. Gene Set Enrichment Analysis

The gene set enrichment analysis (GSEA) program was used to identify gene ontology (GO) terms enriched in *A. minutum* under the induction of nitrogen and phosphorus deficiency [19]. The ranking files required as input for the GSEA program are calculated using the following equation.

Ranking = −log10 FDR × sin log FC

where the values of FDR and FC (fold change) are obtained from the results of the edgeR program analysis [20].

Therefore, unigenes with a high expression level will have a positive score, while unigenes with a low expression level will have a negative score on the ranking file. The rankings for all unigenes were calculated without any cut-off threshold on the FDR values. GO annotation of the *A. minutum* transcriptome from our previous study was used as the set of genes tested by the GSEA program. GO terms annotated to over 500 unigenes and fewer than 15 unigenes were removed from the set of genes tested because GO terms with large annotation sizes are typically involved in overly general biological functions, while GO terms with overly small annotation sizes would not yield results that are statistically significant [20]. GSEA analysis was then performed using a “pre-ranked” method based on 1000 times the permutation of the gene set, and FDR values below 0.25 were considered significant results.

### 3.5. Enrichment Map Visualisation

The “enrichment map” function in the Cytoscape version 3.8.0 program was used to visualize GO terms enriched through GSEA analysis [96,97]. To reduce the complexity of the enrichment map, only GO terms with an FDR threshold value below 0.1 are displayed on this map. Next, GO terms with nearly similar functions were clustered using the “clusterMaker” function, while the “AutoAnnotate” function was used to identify themes for the formed clusters [98]. Finally, the enrichment map is displayed in a “yFiles Organic Layout” layout with manual editing for better visualization.

## 4. Conclusions

In summary, this study provides new insights into the transcriptome changes of *A. minutum* in cultures under nitrogen and phosphorus deficiency, revealing distinct metabolic and cellular responses to these major nutrient deficiencies. The results highlight the significant role of various biological processes, including meiosis, carbon metabolism, ion homeostasis, and post-translational modifications, in regulating dinoflagellate cellular processes. The identification of specific unigenes that regulate these processes provides valuable information for future research in this area. Overall, these findings have implications for our understanding of how dinoflagellates acclimate to changing nutrient conditions and the potential impacts on marine ecosystems and saxitoxin biosynthesis.

## Data Availability

The raw read sequences of transcriptome assembly obtained from Illumina Hiseq4000 (150PE) platform have been deposited in Sequence Read Archive (SRA) under the BioProject id: PRJNA914202.

## Supplementary Materials

The following supporting information can be downloaded at: https://www.mdpi.com/article/10.3390/md21090497/s1, Table S1: Full list of DEGs; Table S2: Gene set enrichment analysis results; Table S3: *A. minutum* unigenes annotation.
